# Supplementary material for: Comprehensive Evaluation of Raw Eating Quality in 81 Sweet Potato (Ipomoea batatas (L.) Lam) Varieties
Source: Foods. 2023 Jan 6;12(2):261. doi: 10.3390/foods12020261 (PMC9858325; doi:10.3390/foods12020261)
Supplement: Supplementary file 1 [file foods-12-00261-s001.zip › Table S1.pdf]

Table S1 The source of eighty-one sweet potato varieties

No.	Varieties	Source	No.	Varieties	Source	No.	Varieties	Source
1	Chuanshu231	Sichuan	28	Luoshu6	Henan	55	Sushu28	Jiangsu
2	Eshu15	Hubei	29	Luosushu18	Henan	56	Sushu29	Jiangsu
3	Eshu17	Hubei	30	Luoxushu9	Henan	57	Sushu23	Jiangsu
4	Eshu19	Hubei	31	Luozishu6	Henan	58	Sushu9	Jiangsu
5	Ezishu13	Hubei	32	Benimasari	Japan	59	Taishu14	Shandong
6	Funingzi3	Fujian	33	Miannanshu10	Sichuan	60	WanA4921	Anhui
7	Fushu604	Fujian	34	Mianshu8	Sichuan	61	Wansu4723	Anhui
8	Futian1	Anhui	35	Mianzishu15	Sichuan	62	Wansu546	Anhui
9	Ganshu6	Jiangxi	36	Nanshu022	Sichuan	63	Wanshu9	Chongqing
10	Guangshu18-10	Guangdong	37	Nanshu88	Sichuan	64	Xinxiang	Zhejiang
11	Guangshu87	Guangdong	38	Nanshu99	Sichuan	65	Xushu18	Jiangsu
12	Guangzishu9	Guangdong	39	Nanzishu018	Sichuan	66	Xushu32	Jiangsu
13	Honghong1	Zhejiang	40	NingB73-8	Jiangsu	67	Xushu37	Jiangsu
14	Jishu29	Shandong	41	Ning C2-6	Jiangsu	68	Xushu44	Jiangsu
15	Jishu33	Shandong	42	Ningzishu10	Jiangsu	69	Xuzishu8	Jiangsu
16	Jishu98	Hebei	43	Ningzishu2	Jiangsu	70	Yunshu678	Shanxi
17	Jiyuan1	Hebei	44	Ningzishu3	Jiangsu	71	Zhanshu16	Guangdong
18	Jinshu69	Zhejiang	45	Ningzishu6	Jiangsu	72	Zhanshu18	Guangdong
19	Liaoshu5	Liaoning	46	Nongdahong	Beijing	73	Zhanshu271	Guangdong
20	Longshu14	Fujian	47	Pushu32	Guangdong	74	Zhanshu407	Guangdong
21	Longshu24	Fujian	48	Qining16	Shandong	75	Zhanzishu2	Guangdong
22	Longshu14	Fujian	49	Qining17	Shandong	76	Zheshu21	Zhejiang
23	Longshu5	Fujian	50	Qining19	Shandong	77	Zheshu27	Zhejiang
24	Longshu9	Fujian	51	Qining26	Shandong	78	Zheshu70	Zhejiang
25	Longzishu9	Fujian	52	Qining37	Shandong	79	Zheshu75	Zhejiang
26	Luoshu10	Henan	53	Shangshu19	Henan	80	Zheshu81	Zhejiang
27	Luoshu14	Henan	54	Sushu25	Jiangsu	81	Zhezishu1	Zhejiang

Note: Only "Benimasari" is from Japan, other varieties are from China.
