# Supplementary material for: Comprehensive Evaluation of Raw Eating Quality in 81 Sweet Potato (Ipomoea batatas (L.) Lam) Varieties
Source: Foods. 2023 Jan 6;12(2):261. doi: 10.3390/foods12020261 (PMC9858325; doi:10.3390/foods12020261)
Supplement: Supplementary file 1 [file foods-12-00261-s001.zip › Table S3.pdf]

Table S3 KMO and Bartlett's Tests of principal component analysis

Kaiser-Meyer-Olkin Measure of Sampling Adequacy.		0.653
Bartlett's Test of Sphericity	Approx. Chi-Square	1738.345
	df	120
	Sig.	0.000
