# Supplementary figures and images for: Comprehensive Evaluation of Raw Eating Quality in 81 Sweet Potato (Ipomoea batatas (L.) Lam) Varieties
Source: Foods. 2023 Jan 6;12(2):261. doi: 10.3390/foods12020261 (PMC9858325; doi:10.3390/foods12020261)

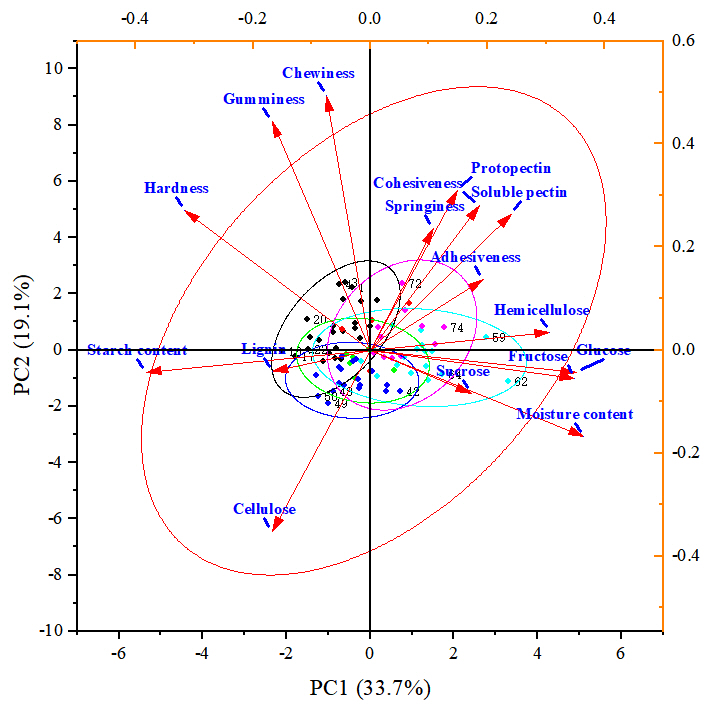

Supplement: Supplementary file 1 [file foods-12-00261-s001.zip › FigureS1. Biplot graph of principal component analysis.jpg]
